# Solid-State Structures and Photoluminescence of Lamellar Architectures of Cu(I) and Ag(I) Paddlewheel Clusters with Hydrogen-Bonded Polar Guests

**DOI:** 10.3390/molecules26216731

**Published:** 2021-11-06

**Authors:** Haruki Inoue, Yuga Yamashita, Yoshiki Ozawa, Toshikazu Ono, Masaaki Abe

**Affiliations:** 1 Graduate School of Science, University of Hyogo, 3-2-1, Kouto, Kamigori-cho, Kobe 678-1297, Japan; ri20e004@stkt.u-hyogo.ac.jp (H.I.); mabe233@gmail.com (Y.Y.); 2 Graduate School of Engineering, Kyushu University, 744 Motooka, Nishi-ku, Fukuoka 819-0395, Japan; tono@mail.cstm.kyushu-u.ac.jp

**Keywords:** hexanuclear paddlewheel clusters, crystal structures, photoluminescence, guest-induced luminescence enhancement, non-covalent interactions

## Abstract

Two hexanuclear paddlewheel-like clusters appending six carboxylic-acid pendants have been isolated with the inclusion of polar solvent guests: [Cu_6_(Hmna)_6_]·7DMF (**1**·7DMF) and [Ag_6_(Hmna)_6_]·8DMSO (**2**·8DMSO), where H_2_mna = 2-mercaptonicotininc acid, DMF = *N*,*N*’-dimethylformamide, and DMSO = dimethyl sulfoxide. The solvated clusters, together with their fully desolvated forms **1** and **2**, have been characterized by FTIR, UV–Vis diffuse reflectance spectroscopy, TG-DTA analysis, and DFT calculations. Crystal structures of two solvated clusters **1**·7DMF and **2**·8DMSO have been unambiguously determined by single-crystal X-ray diffraction analysis. Six carboxylic groups appended on the clusters trap solvent guests, DMF or DMSO, through H-bonds. As a result, alternately stacked lamellar architectures comprising of a paddlewheel cluster layer and H-bonded solvent layer are formed. Upon UV illumination (*λ*_ex_ = 365 nm), the solvated hexasilver(I) cluster **2**·8DMSO gives intense greenish-yellow photoluminescence in the solid state (*λ*_PL_ = 545 nm, *Φ*_PL_ = 0.17 at 298 K), whereas the solvated hexacopper(I) cluster **1**·7DMF displays PL in the near-IR region (*λ*_PL_ = 765 nm, *Φ*_PL_ = 0.38 at 298 K). Upon complete desolvation, a substantial bleach in the PL intensity (*Φ*_PL_ < 0.01) is observed. The desorption–sorption response was studied by the solid-state PL spectroscopy. Non-covalent interactions in the crystal including intermolecular H-bonds, CH⋯π interactions, and π⋯π stack were found to play decisive roles in the creation of the lamellar architectures, small-molecule trap-and-release behavior, and guest-induced luminescence enhancement.

## 1. Introduction

The synthesis of photoluminescent transition-metal complexes with d^6^, d^8^, and d^10^ configurations has attracted widespread interest recently and has been explored extensively [1,2,3,4,5] due to their flexible ability in tuning the emission energy, intensity, and lifetime by molecular design, crystal engineering, and external stimuli such as temperature [6,7,8], pressure [9,10,11], vapors [12,13,14,15,16,17,18,19,20,21,22], and mechanical forces [23,24,25]. These efforts give rise to the essential basis for their applications to various materials such as lighting sources, organic light-emitting diodes, sensors, displays, and imaging devices. In this regard, d^10^ Cu(I) and Ag(I) provide rich photoluminescent complexes due to their flexible coordination number and geometry, and the creation of diverse arrays of solid-state structures ranging from mononuclear to polynuclear complexes and their supramolecular ensembles has been achieved [26,27,28,29,30]. 

Among them, paddlewheel-like hexanuclear Cu(I) and Ag(I) clusters with isomorphous structures [M_6_(Hmna)_6_] or [M_6_(mna)_6_]^6−^ (M = Cu and Ag, H_2_mna = 2-mercaptonicotininc acid) and their derivatives have gathered special attention recently [31,32,33,34,35,36,37,38,39,40,41]. The clusters have been studied from various perspectives. For example, Nomiya et al. determined the crystal structure of a hydrated hexasilver(I) cluster [Ag_6_(Hmna)_6_]·4H_4_O [36]. Subsequently, the crystal structures of [Ag_6_(mna)_6_]^6−^ with 3d transition-metal salts were reported [32,37,40], in which their PL behavior was affected by outer-sphere second transition-metal ions. More recently, Natarajan et al. synthesized [Cu_6_(Hmna)_6_] as well as fully deprotonated [Cu_6_(mna)_6_]^6−^ and partially deprotonated [Ag_6_(Hmna)_2_(mna)_4_]^4−^ clusters as Mn(II) salts via stepwise crystallization and demonstrated their hydrochromic response in the solid state [38]. The same group also addressed the utility of [Ag_6_(mna)_6_]^6−^ of alkaline metal salts for the detection of nitroaromatics in solution by luminescence titration [33]. The carboxylic groups (–COOH) from Hmna^−^ or mna^2−^ ligands appended to the hexanuclear clusters behave as “trap sites” and play a key role in the substrate capture through coordination or H-bonding interactions. Intriguing are antiviral [31] and antimicrobial activity [39] for the hexasilver(I) clusters. 

**Figure 1:**
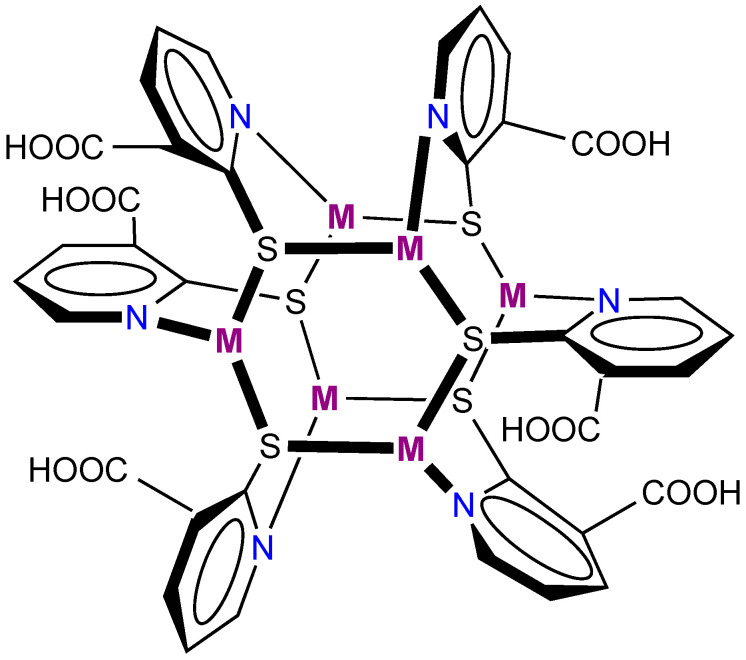
Chemical structures of the [M_6_(Hmna)_6_] clusters, where M = Cu (**1**) and Ag (**2**).

In this work, we succeeded in determining two new crystal structures of copper(I) and silver(I) paddlewheel clusters as solvated forms: [Cu_6_(Hmna)_6_]·7DMF (**1**·7DMF) and [Ag_6_(Hmna)_6_]·8DMSO (**2**·8DMSO). Chemical structures of **1** and **2** are shown in Figure 1. We show that solvated copper cluster **1**·7DMF is, upon UV excitation, a low-energy emitter in the solid state showing near-IR phosphorescence while the silver(I) counterpart **2**·8DMSO vividly emits in the visible region. Significantly, the intense emission observed for both of the solvated clusters is virtually quenched upon complete desolvation, and the resultant tiny emission is then greatly enhanced after gas sorption. 

## 2. Results and Discussion

### 2.1. Synthesis

The synthesis and solid-state structures of **1** and **2** have been reported previously by Nomiya et al. [36] and Kund et al. [38]. Herein, we successfully isolated and structurally characterized their solvated clusters, **1**·7DMF and **2**·8DMSO, in which the solvation number and location were unequivocally determined. The hexacopper(I) cluster with seven DMF guests **1**·7DMF was obtained as orange block-shaped crystals in a test tube by softly layering and slowly diffusing metal- and ligand-precursor solutions (e.g., copper(II) acetate in CH_3_CN and H_2_mna in DMF, at room temperature). The crystalline solid **1**·7DMF, which is insoluble in most organic solvents, gave bright red photoluminescence (PL) under UV illumination. The hexasilver(I) cluster with eight DMSO guests **2**·8DMSO was obtained as pale-yellow block-shaped crystals by recrystallization of **2** from DMSO/Et_2_O at room temperature. Complex **2**·8DMSO, also sparingly soluble in most organic solvents, was photoluminescent in intense greenish yellow in the solid state. 

For both solvated complexes, the solvation number was identified by single-crystal X-ray diffraction analysis (see below). The thermogravimetric (TG) analysis revealed that the solvent loss began in a heating process around room temperature for both crystalline samples and ended at ca. 400 K (Figure S1, Supplementary Materials). On a bench-top experiment, the fully-desolvated solids, **1** and **2**, were prepared by the vacuum-pump method at 353 K. As described later, the intense solid-state PL from both solvated clusters was significantly diminished upon desolvation, whose dramatic PL change is easily recognized by the naked eye. 

### 2.2. Crystal Structures

**Figure 2:**
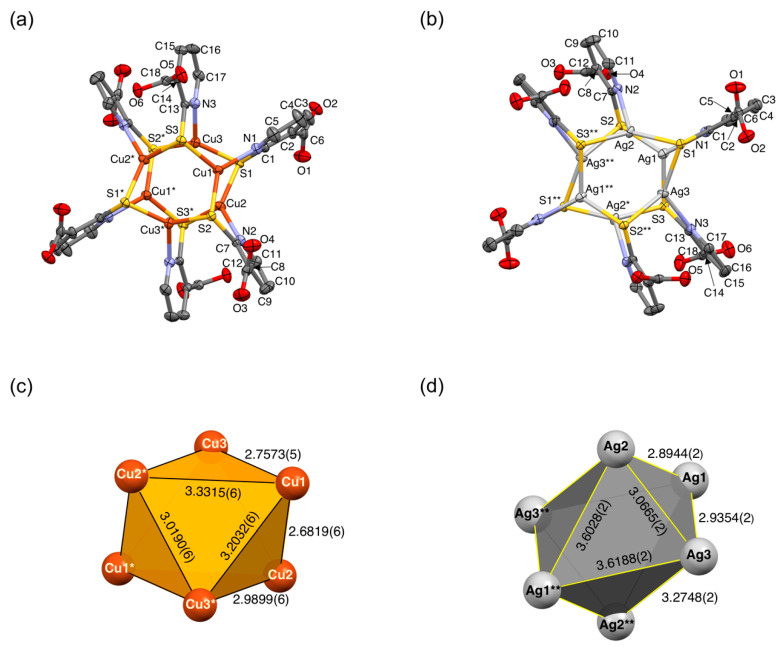
ORTEP drawings of the molecular structures of (**a**) **1**·7DMF and (**b**) **2**·8DMSO at 150 K. Thermal ellipsoids are drawn for non-hydrogen atoms at a 50% probability level. Solvated molecules are omitted for clarity. Octahedral metal cores of (**c**) **1** and (**d**) **2** with intermetallic distances in Å projected along the −3 axis of the virtual molecular symmetry. Atoms labeled with asterisks (* or **) are related to their original atoms by a crystallographic inversion center located in the middle of the molecule.

The detailed crystallographic data on **1**·7DMF and **2**·8DMSO are given in Table 1, and the molecular structures of **1** and **2** are shown in Figure 2. Selected interatomic distances and bond angles are collected in Table 2. 

**1**·7DMF and **2**·8DMSO crystallize in a triclinic space group *P*−1. The cluster molecules are located on an inversion center in the unit cell, in which a half of the molecule (three metal ions and ligands) is crystallographically independent. We found that the crystal lattice constant of **2**·8DMSO was virtually consistent with that reported previously for [Ag_6_(Hmna)_6_] [36]. Both clusters had six-bladed paddlewheel structures in which a 3-fold rotoinversion axis (−3) penetrates the midpoint of the paddlewheel (Figure 2a,b). Each Cu(I) or Ag(I) center adopts a trigonal-planar, distorted three-coordinate geometry with a {NS_2_} donor set. A total of six carboxylic groups (–COOH) per cluster were oriented in an alternate αβαβαβ conformation with respect to the paddlewheel planes, affording trigonally-oriented three –COOH arms on each side.

Six M(I) centers form highly distorted octahedral cores (Figure 2c,d) due to unsymmetrical N,S-bridging mode of Hmna^−^ and the crystal packing, thus providing inequivalent metal–metal separations. For **1**, the Cu⋯Cu separations ranged from 3.0190(6) to 3.3315(6) Å for triangles perpendicular to the −3 axis, whereas the others showed shorter separations ranging from 2.6819(6) to 2.9899(6) Å (Figure 2c and Table 2). These Cu⋯Cu separations were comparable to those reported previously such as neutral [Cu_6_(Hmna)_6_] [38] and hexaanionic [Cu_6_(mna)_6_]^6−^ clusters in [Mn(H_2_O)_6_][Mn_2_(H_2_O)_6_][Cu_6_(mna)_6_]·6H_2_O and [Mn_4_(OH)_2_(H_2_O)_10_][Cu_6_(mna)_6_]·8H_2_O [38]. Similar to **1**, cluster **2** (Figure 2d and Table 2) also showed an unsymmetrical structural feature in the {Ag_6_} skeleton where the Ag⋯Ag separations were identical to those of a series of [Ag_6_(mna)_6_]^6−^ clusters in the literature [32,33,37,39,40].

**Figure 3:**
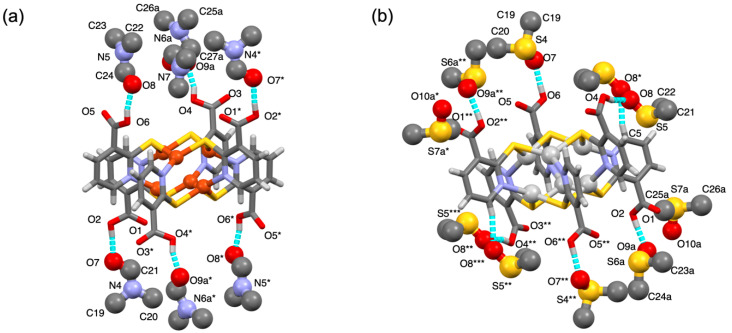
Views of the molecular structures with H-bonded guests in (**a**) **1**·7DMF and (**b**) **2**·8DMSO. Hexanuclear paddlewheels (**1** and **2**) and solvent guest molecules (DMF and DMSO) are depicted in stick and ball-and-stick models, respectively. H-bonds are shown in broken cyan lines. Interatomic distances for H-bonds (*d*_O⋯O_, *d*_C⋯O_, Å) are as follows. For **1**·7DMF, O2⋯O7 = 2.555, O6⋯O8 = 2.552, and O4⋯O9a = 2.534. For **2**·8DMSO, O2⋯O9a = 2.546, O4⋯O8 = 2.546, O6⋯O7 = 2.556, and C5⋯O8* = 3.182. Color codes: Cu, brown; Ag, light gray; S, yellow; O, red; N, blue; C; gray; H, pale gray. Hydrogen atoms of solvent molecules are omitted for clarity. Symmetry codes: (*) 1 − *x*, 1 − *y*, 1 − *z*; (**) 1 − *x*, 2 − *y*, 1 − *z*; (***) *x*, 1 + *y*, *z*.

Solvent guest molecules located around the paddlewheel clusters are shown in Figure 3. Six guests (per cluster) were trapped to the –COOH side arms from the paddlewheel cluster through intermolecular H-bonds. In **1**·7DMF (Figure 3a), six of the seven DMF molecules per cluster was H-bonded to COOH via COOH⋯O=C(H)- (DMF) contact with *d*_O⋯O_ = 2.53–2.56 Å, while the remaining DMF molecule (not shown) was located on the crystallographic –3 axis without any assistance by intermolecular contacts. In **2**·8DMSO (Figure 3b), six DMSO guests were H-bonded to COOH via COOH⋯O=S (DMSO) contact with *d*_O⋯O_ = 2.54–2.56 Å. Two of these six DMSO had an additional contact through H-bonds from the S=O group (labeled as O8* and O8*** atoms in Figure 3b) to the pyridyl moiety in Hmna^−^ with *d*_C⋯O_ = 3.18 Å. The remaining two solvent molecules were encapsulated in the cavity without any significant contacts. 

**Figure 4:**
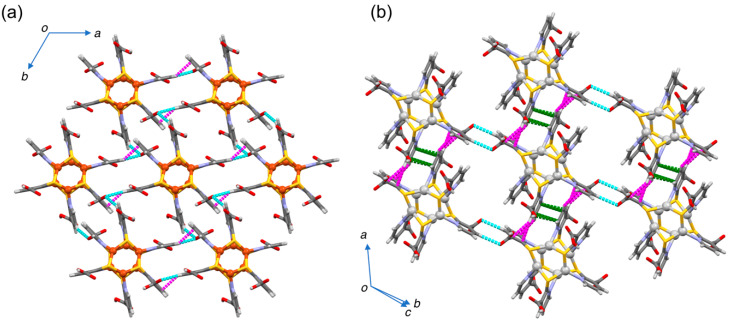

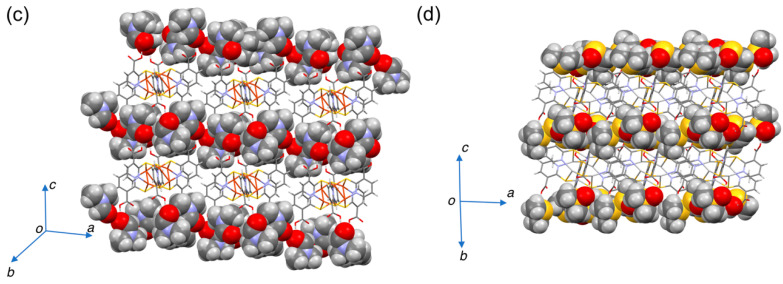
Crystal packing diagrams of (**a**) **1**·7DMF viewed along the *c*-axis and (**b**) **2**·8DMSO viewed along the [0 −1 1] direction. Solvent guests were omitted for clarity. Intermolecular CH⋯O H-bonds, CH⋯π interactions*,* and π⋯π stacks are represented as cyan, magenta, and green broken lines, respectively. Alternate lamellar stacking in (**c**) **1**·7DMF and (**d**) **2**·8DMSO comprising of the paddlewheel layer (shown as a stick model) and the solvent–guest layer (shown as a space-fill model). Color codes: Cu, brown; Ag, light gray; S, yellow; O, red; N, blue; C, gray; H, pale gray.

It was found that the clusters formed 2D sheet motifs, as shown in Figure 4. In **1**·7DMF (Figure 4a), a 2D monolayer sheet was comprised of hexacopper(I) paddlewheels held together by multiple intermolecular non-covalent interactions such as CH⋯O and CH⋯*π* interactions, while in **2**·8DMSO (Figure 4b), it can be seen as intermolecular CH⋯O H-bonds and *π⋯π* contacts. The solvent guest molecules form layers on both sides of the paddlewheel sheet. As a result, the lamellar structure comprises an alternate stack of two distinct layers (Figure 4c,d). 

### 2.3. Solid-State Spectroscopy and DFT Calculations 

UV–Vis diffuse reflectance spectra of desolvated clusters **1** and **2** exhibited shoulders at *ca.* 450 and 420 nm, respectively (Figure S2, Supplementary Materials). DFT calculations on **1** (Figure S3, part a, Supplementary Materials) revealed that the HOMO (162A_u_) could be attributed to a Cu-3d/S-2p orbital in the {Cu_6_S_6_} core with a non-bonding character, while the LUMO (163A_u_) was localized on the ligand pyridyl ring with a π* character. Therefore, the observed absorption shoulder was assigned to a copper/sulfur-to-ligand charge-transfer transition. For **2** (Figure S2, part b), the HOMO (189A_g_) was comprised of Ag-4d/S-2p orbital, while the LUMO (190A_g_) was localized on the {Ag_6_S_6_} core (Figure S2, part b). An unoccupied π* ligand orbital LUMO+1 (190A_u_) was also found at a slightly higher level to the LUMO. Accordingly, the absorption shoulder for **2** was tentatively assigned to a {Ag_6_S_6_} cluster-centered transition and/or a silver/sulfur-to-ligand charge-transfer transition. 

In the FTIR spectra (Figure S4 and Table S1, Supplementary Materials), the presence of solvent molecules in **1**·7DMF and **2**·8DMSO [42] was clearly confirmed. Moreover, vibration peaks ascribed to C=O and C–OH in the COOH pendants for solvated solids were observed at ca. 1710 and ca. 1390 cm^−1^, respectively. We observed that these frequencies were apparently affected by desolvation, but the direction of the frequency shift depended on the compounds. This ambiguity probably comes from complexity arising from crystal-packing change or amorphization occurring in the solvent desorption process. In order to address this issue, a powder X-ray diffraction study is required, which is in progress in our laboratory.

### 2.4. Solid-State Photoluminescence 

Solvated hexacopper(I) complex **1**·7DMF exhibited PL at *λ*_PL_ = 765 nm with a microsecond-order lifetime (*τ*_PL_ = 5.9 μs), suggesting that the PL is phosphorescence. On the basis of our current DFT calculations, this PL was assigned to the Cu/S-to-ligand charge-transfer triplet excited state. It is noted that the excited-state nature for the paddlewheel-type hexacopper(I) clusters with N^S bridging ligands such as **1** varied depending on the nature of the ligands. For example, the phosphorescence observed for [Cu_6_(6-mpyt)_6_] (6-mpyt^−^ = 6-methyl-2-pyridinethiolate) could be attributed to a cluster-centered lowest triplet (^3^CC) excited state [43]. Our recent report [6] showed that the [Cu_6_(Me-bimt)_6_] complex (Me-bimt^−^ = *N*-methylbenzimidazolate) exhibited a near-IR PL band (*λ*_PL_ = 876 nm), which was also assigned to the ^3^CC (Cu_6_S_6_) excited state. In contrast, a ligand-to-metal charge-transfer (LMCT) mixed with a cluster-centered triplet state was described for [Cu_6_(mtc)_6_] (mtc^−^ = di-*n*-poropylmonothiocarbamate) [4,5]. As for **1**, the low-lying ligand π* orbital in Hmna^−^, which corresponds to LUMO, played a decisive role in its phosphorescence. 

For solvated hexasilver(I) complex **2**·8DMSO, the observed PL (*λ*_PL_ = 545 nm, *τ*_PL_ = 9.3 μs, and *Φ*_PL_ = 0.17) was ascribable to the ^3^CC (Ag_6_S_6_) excited state and/or a Ag/S-to-ligand charge-transfer triplet excited state, as predicted by our DFT calculations. Here, the contribution of the ^3^CC excited state to phosphorescence may be rationalized by energy-lowering of the CC orbital (Ag-5s,p/S-3s,p) to become LUMO, which is located slightly below the ligand π* orbitals in energy (Figure S3, part b). For reference, the PL from the hexaanionic [Ag_6_(mna)_6_]^6−^ complexes with various cationic salts was assigned to LMCT mixed with the CC excited state [32,33,40]. 

In sharp contrast to intense PL for the above-mentioned solvated clusters, desolvated **1** and **2** produced significantly weakened PL with *Φ*_PL_ < 0.01 (Table 3). It was also observed that the desolvation allowed the PL lifetimes to be shortened. 

### 2.5. Vapochromic Luminescence

**Figure 5:**
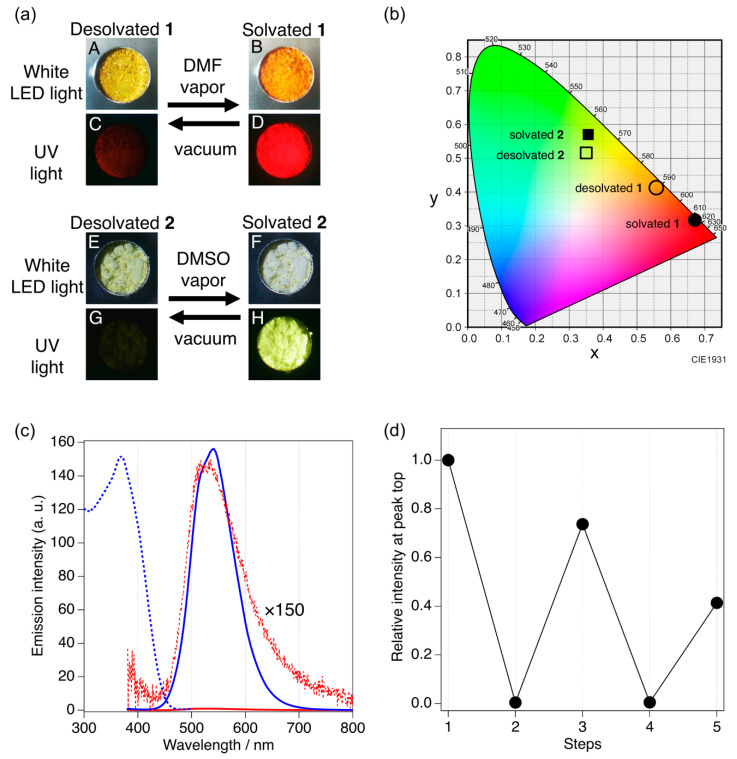
Solid-state vapochromic response. (**a**) Photographic images showing the color change for solid samples **1** (panels A to D) and **2** (panels E to H) under white LED and UV lights. (**b**) Chromaticity color coordinate plots for solvated and desolvated **1** and **2** in the CIE1931 color space [44]. (**c**) Solid-state PL spectra (298 K, *λ*_ex_ = 365 nm) of desolvated **2** (red solid curve) with its 150-times-magnified trace (red dots) and those under a DMSO atmosphere kept for 24 h (blue solid curve). An excitation profile (monitored at 545 nm) is also shown (blue dots). (**d**) The PL intensity change during two cycles for desorption and sorption procedures for **2**·8DMSO (monitored by the intensity at the peak top of PL). Step 1: PL peak intensity of **2**·8DMSO. Steps 2–5: Relative PL peak intensity monitored at desolvated (steps 2 and 4) and solvated states (steps 3 and 5).

Vapochromic response was studied by solid-state PL spectroscopy, as shown in Figure 5. Desolvated **1** and **2** used for the vapochromic experiments were prepared by vacuum pumping of **1**·7DMF and **2**·8DMSO, respectively, for 0.5 h at 353 K. The vapor sorption was then achieved by standing the desolvated samples under the atmosphere of saturated DMF or DMSO vapor for 24 h at 296 K.

Under a white LED light, the color of desolvated **1** was yellow (panel A in Figure 5a), which changed into orange under the DMF vapor (panel B). For the same sample as above, the PL of desolvated **1** was faint red (panel C), but the PL was then remarkably enhanced to vivid red after DMF sorption (panel D). For desolvated **2**, the pale-yellow solid under the white light (panel E) became white under the DMSO vapor (panel F). Upon UV exposure, an almost invisible PL from desolvated **2** (panel G) became intensely brightened with a vivid greenish yellow upon sorption of the DMSO vapor (panel H). We confirmed that the initial faint PL for the desolvated solids was completely regenerated from the vividly-colored gas-sorbed solids by conducting the vacuum pumping at elevated temperature (0.5 h, 353 K). Chromaticity color coordinate plots [44] (Figure 5b) also characterize two distinct states (“solvated” and “desolvated”) for **1** and **2**. 

The solid-state PL spectroscopy (Figure 5c and Figure S5 for **2** and **1**, respectively) revealed that the very weak luminescence of desolvated **2** (red solid curve with red dots for the magnified trace) was significantly enhanced after the desolvated solid was stood under a DMSO atmosphere (blue solid curve, Figure 5c). The enhanced PL spectrum was no longer changed under continuous exposure to the DMSO vapor, suggesting that the sorption was completed within 24 h. Desolvated **1** also showed a similar behavior toward DMF in the PL spectra, in which ca. 40-times enhancement was observed under DMSO (Figure S5). 

Such a vapor-induced PL enhancement occurs by trapping guest molecules to form intermolecular H-bonds as shown by X-ray crystallography. The guest trapping through H-bonds may restrict the freedom of molecular motions or thermal vibrations in the crystalline state, resulting in suppression of the probability of the non-radiative decay of the excited states and, as a consequence, the solid-state luminescence is enhanced. In the literature, issues on the effects of non-covalent interactions to solid-state luminescence have been addressed for aggregation-induced emission systems [45,46,47]. 

Finally, desorption–sorption cycles were examined for **2**·8DMSO by probing the PL intensity. A moderate degree of reversibility was shown (Figure 5d). 

An appealing advantage has been demonstrated for complexes **1** and **2** as solid-state turn-on detectors toward polar VOCs by use of visible (Ag_6_) or near-IR (Cu_6_) optical windows. Further study is in progress on the gas-sorption/desorption ability of these paddlewheel clusters in terms of gas selectivity, response speed, and reproducibility for multiple operations.

## 3. Materials and Methods 

### 3.1. Materials

Chemical reagents and solvents used in this study were purchased from TCI, WAKO, and Nakalai, and used as received. All the synthetic reactions in this study were performed under an Ar atmosphere unless otherwise stated. Complex **2** was prepared by the reported method [36]. 

### 3.2. Physical Measurements

Fourier transform infrared (FTIR) spectra were obtained on a JASCO FT/IR-4000 with the attenuated total reflectance (ATR) method for the solid samples. Solid-state UV–Vis diffuse reflectance UV–Vis spectra were measured by a HITACH U-3500 UV–Vis-NIR spectrometer with an integrating sphere at 296 K. Thermogravimetry and differential thermal analyses (TG-DTA) were performed on a Rigaku Thermoplus EV02. Elemental analysis was carried out by the Laboratory for Organic Elemental Microanalysis, Kyoto University.

### 3.3. Photoluminescence and Photophysical Measurements

Solid-state PL spectra were measured by a Hamamatsu PMA-11 (C7473-46) spectrometer (190 to 900 nm). The crystalline samples were placed in a Linkam THMS 600 temperature-controlled (78 to 293 K) microscope stage. A Panasonic UJ-30 UV-LED system was used as an excitation light source (*λ*_PL_ = 365 nm). The PL from the sample was introduced to the optical fibers of the spectrometer through a microscope. The excitation spectra for solids were measured by a JASCO FP-8500 spectrofluorometer with an FPA-810 powder sample cell block. The absolute PL quantum yields (PLQYs) were measured by a Hamamatsu C9920-02 system with an integrating sphere. The PL lifetimes were measured on a Hamamatsu Quantaurus-Tau C11367-02 fluorescence lifetime spectrometer. 

### 3.4. Synthesis of [Cu_6_(Hmna)_6_]·7DMF (***1***·7DMF)

A DMF solution (12.5 mL) containing 2-mercaptonicotinic acid (H_2_mna, 0.186 g, 1.20 mmol) was prepared as “Solution A”. Separately, a CH_3_CN solution (13.0 mL) of copper(II) acetate dihydrate Cu(CH_3_COO)_2_·2H_2_O (0.240 mg, 1.20 mmol) was prepared as “Solution B”. In a test tube, Solution A (4.0 mL) was placed at the bottom, and to this was carefully layered a buffer solution (DMF/CH_3_CN = 1:1, 4 mL) and then layered on top Solution B (4 mL). The test tube was allowed to stand in the dark at 293 K to grow block-shaped orange crystals **1**·7DMF, from which single crystals suitable for X-ray structural determination were found. Yield: 0.035 g (9%). Anal. Calcd. for C_36_H_24_Cu_6_N_6_O_12_S_6_ (an unsolvated form after complete drying by pumping): C, 33.10; H, 1.85; N, 6.43%. Found: C, 33.10; H, 1.99; N, 6.67%. FTIR (ATR, cm^−1^): 1706 (s, *ν*(C=O)), 1391 (s, *ν*(C–OH)). 

### 3.5. Synthesis of [Ag_6_(Hmna)_6_]·8DMSO (***2***·8DMSO) 

A DMSO solution (40 mL) of **2** (1.06 g) was prepared in a three-necked round-bottom flask and placed in an ice-cooled water bath. To this was slowly added Et_2_O (170 mL) with vigorous stirring to instantly precipitate a white solid. After vigorous stirring for 2 h, the white solid, which was photoluminescent in a vivid greenish yellow under UV light, was collected by filtration, washed with Et_2_O, and dried under vacuum. Yield, 0.77 g (53%). FTIR (ATR, cm^−1^): 1716 (s, *ν*(C=O)), 1388 (s, *ν*(C–OH)). 

Single crystals of **2**·8DMSO suitable for X-ray crystallography were obtained by slow vapor diffusion of Et_2_O into a DMSO solution of **2** for 24 h at 293 K. 

### 3.6. Synthesis of Desolvated [Cu_6_(Hmna)_6_] (***1***) and [Ag_6_(Hmna)_6_] (***2***)

These compounds were prepared by vacuum drying of solvated clusters, **1**·7DMF and **2**·8DMSO, for 0.5 h at 353 K. Complete desolvation was confirmed by TG-DTA, showing the absence of weight loss due to residual solvent desorption and elemental analysis. Complex **1**: Anal. Calcd. for C_36_H_24_Cu_6_N_6_O_12_S_6_: C, 33.10; H, 1.85; N, 6.43%. Found: C, 33.10; H, 1.99; N, 6.67%. FTIR (ATR, cm^−1^): 1725 (s, *ν*(C=O)), 1396 (s, *ν*(C–OH)). Complex **2**: Anal. Calcd. for C_36_H_24_Ag_6_N_6_O_12_S_6_: C, 27.50; H, 1.54; N, 5.35%. Found: C, 27.34; H, 1.66; N, 5.27%. FTIR (ATR, cm^−1^): 1704 (s, *ν*(C=O)), 1389 (s, *ν*(C–OH)). 

### 3.7. X-ray Crystallography

Single-crystal X-ray diffraction analysis for **1**·7DMF and **2**·8DMSO was performed on a Rigaku Rapid image plate diffractometer at 150 K using Mo-K*α* radiation (*λ* = 0.71073 Å). The crystal was attached to a thin Nylon loop and kept under a stream of cold N_2_ gas from a He gas-expansion cryostat. The structures were solved by a direct method with SHELXT-2018/2 [48] and refined by using the full-matrix least-squares method based on *F*^2^ with the SHELXL-2018/3 refinement program [49]. An empirical absorption correction was applied. All non-hydrogen atoms, except some disordered oxygen atoms, were refined anisotropically. Hydrogen atoms were located by calculations. All the calculations for structural determination and refinements were carried out using the WinGX [50]. 

For **1**·7DMF, two disordered DMF molecules, DMF-A (comprising of C25, C26, N6, C27, and O9) and DMF-B (comprising of C28, C29, N7 C30, and O10), were seen. The amide group in DMF-A (N6–C27=O9) was located in two orientations where the positions of N6 and C27 were completely disordered. In DMF-B, N7 was located on a crystallographic inversion center and the other atoms were located in two positions. For **2**·8DMSO, two DMSO molecules, DMSO-A (C23, C24, S6, and O9) and DMSO-B (C25, C26, S7, and O10), were disordered. For DMSO-A, the S atom was located above and below the C23–C24–O9 plane with an uneven occupancy factor of 0.9:0.1. The molecule DMSO-B was disordered in two orientations and located with an occupancy factor of 0.8:0.2. For these disordered atoms, the major and minor component atoms were labelled “a” and “b”, respectively, as in S6a and S6b. The minor components, labelled “b”, are omitted in Figure 3 for the sake of clarity. 

### 3.8. DFT Calculations 

DFT calculations were carried out with the SCM ADF2019 suite of programs [51,52]. The initial geometrical parameters for **1** and **2** were taken from the corresponding crystal structures at 150 K, symmetrizing the molecules to adapt *C_i_* symmetry as in the crystal structures, and used for geometrical optimization. The exchange-correlation (XC) energy function of the PW91 method was applied for calculations. The valence shell atomic orbitals of Cu, Ag, S, N, C, and H atoms were described by triple-zeta Slater-type basis sets with two polarization functions (ADF database TZ2P). 

### 3.9. Photophysical Measurements

Completely desolvated samples **1** and **2** for use in the gas-sorption experiments were prepared by vacuum pumping (0.5 h at 353 K) of the corresponding solvated samples **1**·7DMF and **2**·8DMSO, respectively, which were loaded on an aluminum sample holder. The gas sorption experiments were then achieved by standing the desolvated samples under vapor in a Petri dish-like seal container at 298 K. The in situ solid-state PL measurements were carried out in the optical window between 190 and 950 nm while allowing for the solid samples to maintain the vapor-saturated conditions during the PL measurements. The PL spectra were recorded at a constant interval, typically at every 2 h, to trace the time course of the sorption process until the PL spectra reach the saturation level. 

## 4. Conclusions

The paddlewheel-like hexanuclear clusters [Cu_6_(Hmna)_6_] (**1**) and [Ag_6_(Hmna)_6_] (**2**) were isolated as solvated forms **1**·7DMF and **2**·8DMSO. Single-crystal X-ray diffraction analysis revealed the formation of lamellar architectures composed of an alternate stack of two distinct layers: “paddlewheel cluster layer” (supported with intermolecular CH⋯π and π⋯π interactions) and “solvent guest layer”, where DMF or DMSO guests are bound through intermolecular H-bonds to carboxylic pendants from the paddlewheel molecule. The **1**·7DMF and **2**·8DMSO exhibited microsecond-order, intense PL in the solid state in the near-IR and visible wavelength regions, respectively. In contrast, desolvated forms **1** and **2**, prepared by vacuum drying for the solvated forms, were tiny emitters due to vibrational non-radiative decay. Remarkable recovery for intense PL was observed by spontaneous gas sorption. The vapor-induced luminescence enhancement may promise the future development of novel VOC-sensitive solid-state sensors and switching devices. Along this line, the paddlewheel clusters described here, which were synthesized from inexpensive metal and ligand sources commercially available, should be an elaborate molecular motif. Further study is in progress to improve their response ability in terms of sensitivity, selectivity, and response speed in order to develop more advanced materials for sensing VOCs and hazardous chemicals in the atmosphere. 

## Figures and Tables

**Table 1:** Crystallographic and structure refinement data for **1**·7DMF and **2**·8DMSO.

Compound	1·7DMF	2·8DMSO
Formula	C_57_H_73_Cu_6_N_13_O_19_S_6_	C_52_H_72_Ag_6_N_6_O_20_S_14_
Formula Weight	1817.88	2197.21
Crystal Size (mm)	0.56 × 0.53 × 0.35	0.23 × 0.20 × 0.16
Color	orange	pale yellow
Habit	block	block
*T* (K)	150	150
Crystal System	triclinic	triclinic
Space Group	*P*−1 (No. 2)	*P*−1 (No. 2)
*a* (Å)	13.1804(4)	11.7593(3)
*b* (Å)	13.4734(4)	12.8538(3)
*c* (Å)	13.7928(3)	15.3089(4)
*α* (°)	115.960(1)	105.979(1)
*β* (°)	95.795(1)	110.979(1)
*γ* (°)	115.287(1)	102.971(1)
*V* (Å^3^)	1862.13(9)	1937.95(9)
*Z*	1	1
*ρ*_calc_ (g cm^−3^)	1.621	1.883
wavelength, *λ* (Å)	0.71073	0.71073
*θ*_max_ (°)	30.0	30.0
*µ*(*λ)* (mm^−1^)	1.925	1.931
*F*(000)	928	1092
reflns. collected	22034	22814
unique reflns.	10531	10957
*R*_int_	0.033	0.018
Completeness	0.995	0.996
Parameters	486	493
*R*_1_, *wR_2_* (*F*^2^) (*I* > 2*σ*) ^1^	0.052, 0.133	0.023, 0.053
*R*_1_, *wR_2_* (*F*^2^) (all data)	0.056, 0.138	0.029, 0.057
*GOF*	1.21	1.106
*ρ*_max_, *ρ*_min_ (e Å^−^^3^)	1.23, −0.79	1.10, −0.41
CCDC	2052413	2052414

^1^$\;R_{1}=\sum \left|\left|F_{o}\right|-\left|F_{c}\right|\right|/\sum \left|F_{o}\right|;\;wR_{2}={\left\{\sum \left[w\left(F_{o}^{2}-F_{c}^{2}\right)\right]/\sum \left[w\left(F_{o}^{2}\right)\right]\right\}}^{1/2}$. $\;w=1/\left[\sigma^{2}\left(F_{o}^{2}\right)+{\left(aP\right)}^{2}+bP\right].\;P=\left[2F_{c}^{2}+max\left(F_{o}^{2},0\right)\right]/3$, where *a* = 0.0373 and *b* = 5.3412 for **1**·7DMF and *a* = 0.0264 and *b* = 0.7341 for **2**·8DMSO.

**Table 2:** Selected interatomic distances (Å) and bond angles (°) for **1**·7DMF and **2**·8DMSO at 150 K.

**1**·7DMF			
Cu1⋯Cu2 ^*^	3.3315(6)	Cu1⋯Cu2	2.6819(6)
Cu1⋯Cu3 ^*^	3.2032(6)	Cu1⋯Cu3	2.7573(5)
Cu2⋯Cu3	3.0190(6)	Cu2⋯Cu3 ^*^	2.9899(6)
Cu1—N1	2.006(3)	Cu2—S3 ^*^	2.2696(8)
Cu1—S2	2.2300(8)	Cu3—N3	2.014(3)
Cu1—S3	2.2548(9)	Cu2—S3 ^*^	2.2696(8)
Cu2—N2	2.014(3)	Cu3—S1	2.2467(8)
Cu2—S1	2.2179(8)		
			
N1—Cu1—S2	129.66(9)	S3—Cu1—S1	120.87(3)
S2—Cu1—S3	106.80(3)	N1—Cu1—Cu3 ^*^	152.81(9)
S2—Cu1—S3	106.80(3)	S2—Cu1—Cu3 ^*^	44.89(2)
N1—Cu1—S1	57.89(8)	S3—Cu1—Cu3 ^*^	89.21(2)
S2—Cu1—S1	120.43(3)		
			
**2**·8DMSO			
Ag1⋯Ag2 ^**^	3.6028(2)	Ag1⋯Ag2	2.8944(2)
Ag1⋯Ag3 ^**^	3.6188(2)	Ag1⋯Ag3	2.9354(2)
Ag2⋯Ag3	3.0665(2)	Ag2⋯Ag3 ^**^	3.2748(2)
Ag1—N1	2.2664(15)	Ag2—S3 ^**^	2.5037(5)
Ag1—S2	2.4831(4)	Ag3—N3	2.2950(15)
Ag1—S3	2.4852(5)	Ag3—S1	2.4592(5)
Ag2—N2	2.3132(15)	Ag3—S2 ^**^	2.5042(5)
Ag2—S1	2.4711(4)		
			
N1—Ag1—S2	124.56(4)	S1—Ag2—S3 ^**^	124.245(15)
N1—Ag1—S3	122.20(4)	N3—Ag3—S1	127.61(4)
S2—Ag1—S3	108.166(15)	N3—Ag3—S2 ^**^	101.66(4)
N2—Ag2—S1	126.17(4)	S1—Ag3—S2 ^**^	123.507(15)
N2—Ag2—S3 ^**^	101.93(4)		

Symmetry codes: (*) 1−*x*, 1−*y*, 1−*z*; (**) 1−*x*, 2−*y*, 1−*z*.

**Table 3:** Solid-state photophysical data (298 K) of paddlewheel clusters in the solvated (**1**·7DMF and **2**·8DMSO) and desolvated (**1** and **2**) forms (*λ*_ex_ = 365 nm).

Compound	*λ*_PL_*^a^*/nm	*τ*_PL_*^b^*/µs	*Φ*_PL_ *^c^*	Luminescence Color
**1**·7DMF	765	5.9	0.38	red
**1** (desolvated)	710	0.65	<0.01	faint red
**2**·8DMSO	545	9.3	0.17	vivid greenish yellow
**2** (desolvated)	548	0.05	<0.01	faint yellow

*^a^* Photoluminescence peak wavelength. *^b^* Photoluminescence lifetime. *^c^* Absolute photoluminescence quantum yield.

## Data Availability

CCDC 2052413 and 2052414 contain the supplementary crystallographic data for this paper. These data can be obtained free of charge via www.ccdc.cam.ac.uk/data_request/cif, or by emailing data_request@ccdc.cam.ac.uk, or by contacting The Cambridge Crystallographic Data Center, 12 Union Road, Cambridge CB2 1EZ, UK; fax: +44-1223-336033.

## Supplementary Materials

The following are available online, Figure S1: Thermogravimetric traces for **1**·7DMF and **2**·8DMSO; Figure S2: Diffuse reflectance UV–Vis spectra for desolvated **1** and **2**; Figure S3: Frontier MOs of **1** and **2** obtained by geometry optimized DFT calculations; Figure S4: FTIR spectra of **1**·7DMF and **2**·8DMSO and their desolvated **1** and **2**; Table S1: Selected FTIR data; Figure S5: Solid-state PL spectra of desolvated **1** and DMF-solvated **1**.

## Sample Availability

Samples of the compounds are available from the authors.
