# Supplementary material for: Solid-State Structures and Photoluminescence of Lamellar Architectures of Cu(I) and Ag(I) Paddlewheel Clusters with Hydrogen-Bonded Polar Guests
Source: Molecules. 2021 Nov 6;26(21):6731. doi: 10.3390/molecules26216731 (PMC8587135; doi:10.3390/molecules26216731)
Supplement: Supplementary file 1 [file molecules-26-06731-s001.zip › molecules-1386550-supplementary.pdf]

Supporting Information

Solid-State Structures and Photoluminescence of Lamellar Architectures of Cu(I) and Ag(I) Paddlewheel Clusters with Hydrogen-Bonded Polar Guests

Haruki Inoue,[1] Yuga Yamashita,[1] Yoshiki Ozawa,*[1] Toshikazu Ono,[2] and Masaaki Abe*[1]

[1]*Graduate School of Science, University of Hyogo, 3-2-1 Kouto, Kamigori-cho, Hyogo 678-1297, Japan.*

[2]*Graduate School of Engineering, Kyushu University, 744 Motooka, Nishi-ku, Fukuoka 819-0395, Japan.*

Contents

Figure S1. Thermogravimetric traces of **1**·7DMF and **2**·8DMSO. S-2

Figure S2. Diffuse reflectance UV–Vis spectra for desolvated **1** and **2**. S-2

Figure S3. Frontier MOs for **1** and **2** obtained by geometry optimized DFT calculations. S-3

Figure S4. FTIR spectra of (a) **1**·7DMF and desolvated **1** and (b) **2**·DMSO and desolvated **2**. S-4

Table S1. Selected FTIR data. S-4

Figure S5. Solid-state PL spectra of desolvated **1** and DMF-solvated **1**. S-5

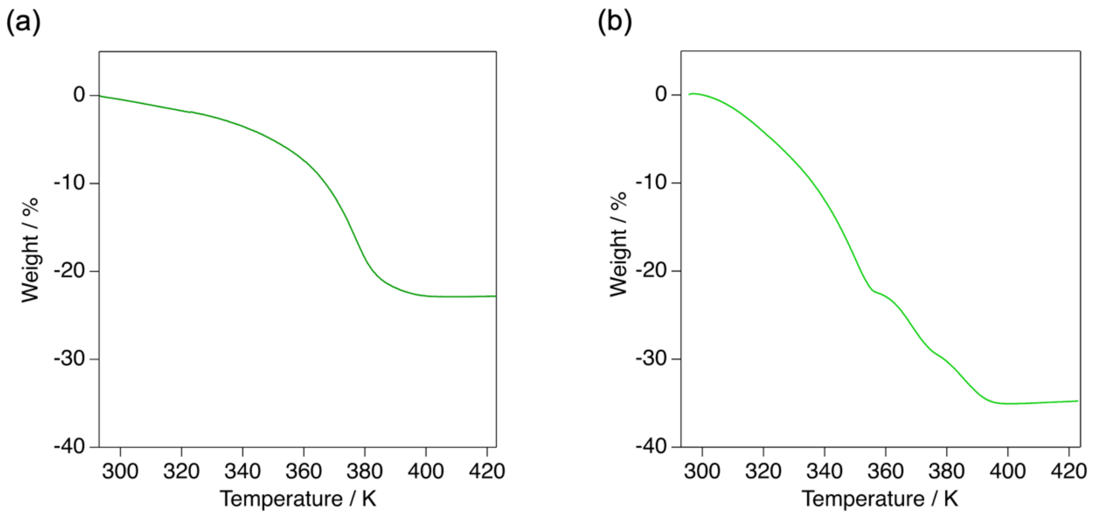

Figure S1. Thermogravimetric traces for (a) **1**·7DMF and (b) **2**·8DMSO recorded in a heating process under N_2 from 293 to 423 K.

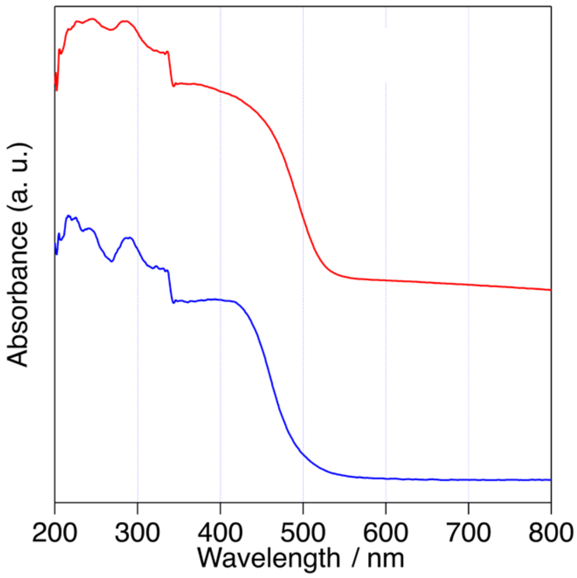

Figure S2. Diffuse reflectance UV–Vis spectra (298 K) of desolvated **1** (red) and **2** (blue). An absorbance artifact located at 340 nm is attributed to the light-source change from WI to D_2.

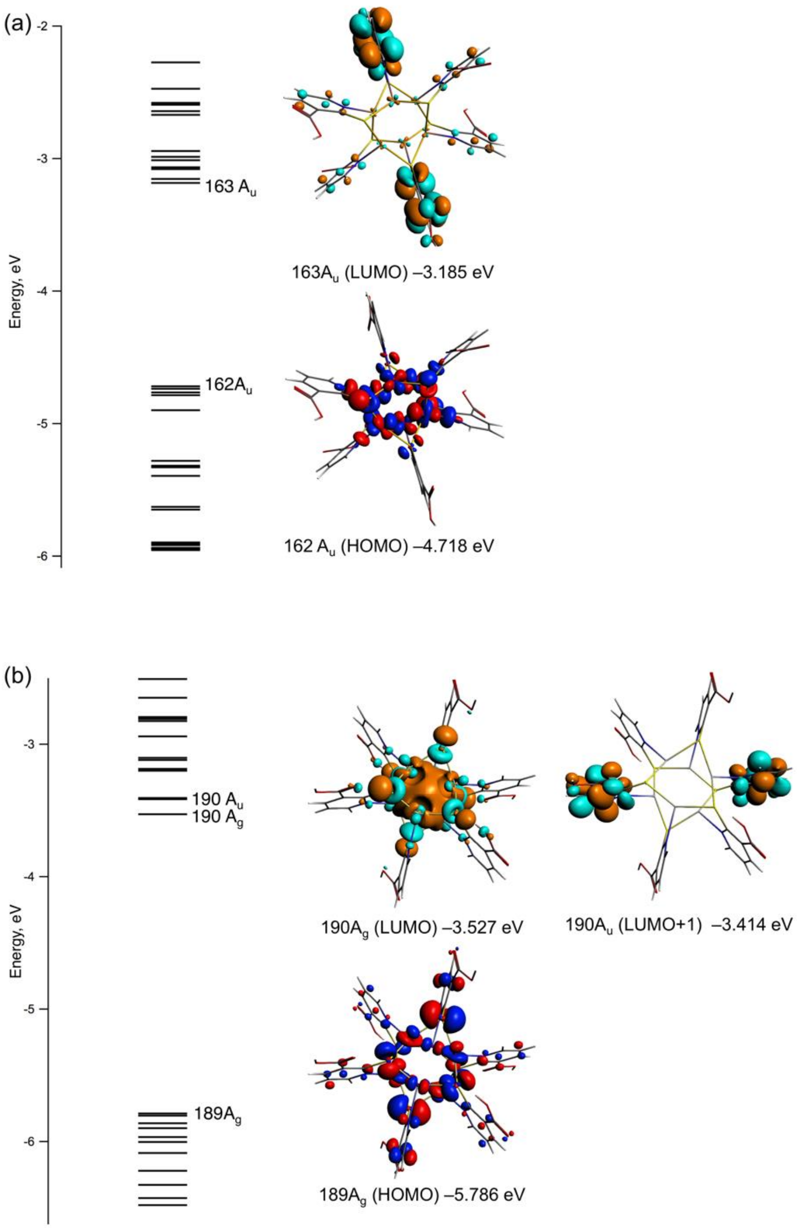

Figure S3. Frontier MOs for (a) **1** and (b) **2** obtained by geometry optimized DFT calculations.

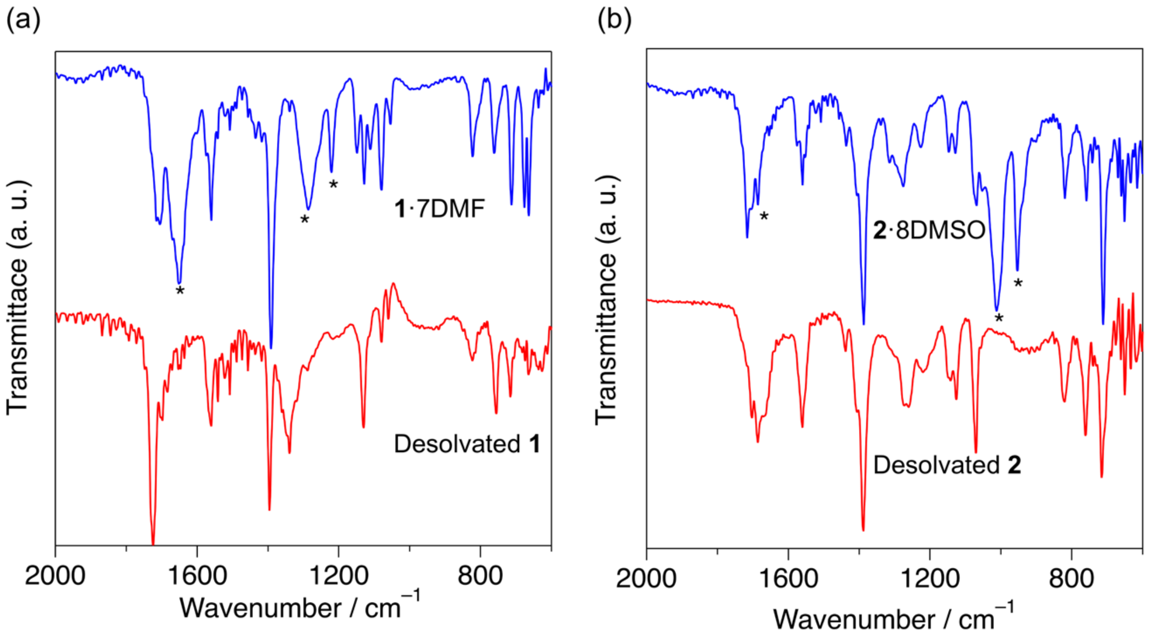

Figure S4. FTIR spectra (ATR, 298 K) of (a) **1**·7DMF (blue) and desolvated **1** (red) and (b) **2**·8DMSO (blue) and desolvated **2** (red). Peaks marked with asterisks (*) are attributed to DMF or DMSO molecules.

Table S1. Selected FTIR data (cm^{-1})

Compounds	ν(C=O)	ν(C–OH)	Solvent molecules
**1**•7DMF	1706 (s)	1391 (s)	1650 (s), 1287 (m), 1221 (w)
**1** (desolvated)	1725 (s)	1396 (s)	–
**2**•8DMSO	1716 (s)	1388 (s)	1684 (m), 1013 (s), 954 (m)
**2** (desolvated)	1704 (s)	1389 (s)	–

s = strong, m = medium, w = weak

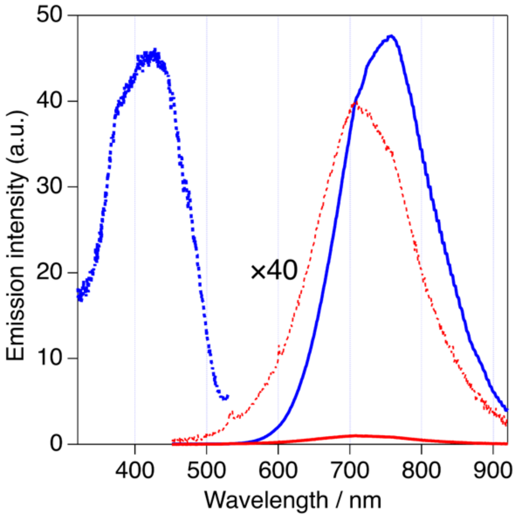

Figure S5. Solid-state PL spectra (298 K, λ_{ex} = 365 nm) of desolvated **1** (red solid curve; a 40-times-magnified trace is depicted by red dots) and DMF-sorbed **1** (blue solid curve) with the excitation spectrum (blue dots). The excitation spectrum was monitored with the PL intensity at 625 nm, rather than at the PL peak top (765 nm), due to limitation for the accessible wavelength range for the spectrofluorometer (up to 700 nm).
